# Our New Stove

**Published:** 1876-12

**Authors:** 


### *OUR NEW STOVE.*

Well, we have given our stove a pretty fair test and now we think we understand it. How to heat our great office has been a serious problem—as, indeed this question of heating houses and stores and apartments, must always be in our variable climate. But we believe we have solved it. This new stove is the largest size—No. 4—which the "Open Stove Ventilating Company," of 107 Fulton Street, make. Our room is some 60 feet long, 20 wide and 14 high. Last winter it took two of those insufferable nuisances—the self-feeders—to heat it, and even then we were either roasted or frozen most of the time. Now we have a delightful temperature with our single stove, and do not consume as much coal as we did in either one of the wretched self-feeders, which we have pushed into a corner and would be glad to give away. A friend who has seen our stove in operation bought one of the same kind, a size smaller, for the sitting-room of his house on Twenty-sixth Street. He is the most enthusiastic person we have seen. He says his new stove heats an entire floor of three rooms, even with a window dropped six inches for ventilation, and that no heating device could be less troublesome. He puts coal on only twice a day—at 7 o'clock, morning and evening—and rarely uses the blower. He tells us to send everybody to see it, and we extend the same invitation as to ours. We think this "Fire on the Hearth" open stove, the pleasantest and best heater we ever saw. It heats the room at remote points as perfectly as in its vicinity, and ventilates it at the same time. So it is healthy as well as comfortable and economical. After we have tested it at zero temperature we will speak of it again, and we don't believe we shall have any cause to complain of it.

